# In vitro and in vivo identification of ABCB1 as an efflux transporter of bosutinib

**DOI:** 10.1186/s13045-015-0179-4

**Published:** 2015-07-07

**Authors:** Sara Redaelli, Pietro Perini, Monica Ceccon, Rocco Piazza, Roberta Rigolio, Mario Mauri, Frank Boschelli, Athina Giannoudis, Carlo Gambacorti-Passerini

**Affiliations:** Department of Health sciences, University of Milano-Bicocca, S.Gerardo Hospital, Monza, Italy; Department of Surgery and Translational Medicine, University of Milano-Bicocca, Monza, Italy; Department of Oncology, Pfizer Research, Pearl River, New City, NY USA; Institute of Translational Medicine, Molecular and Clinical Cancer Medicine, University of Liverpool, Liverpool, UK

**Keywords:** CML, TKI, Drug transporters, Resistance

## Abstract

**Background:**

Bosutinib is a recently approved ABL inhibitor. In spite of the well-documented effectiveness of BCR-ABL inhibitors in treating chronic myeloid leukemia, development of resistance is a continuous clinical challenge. Transporters that facilitate drug uptake and efflux have been proposed as one potential source of resistance to tyrosine kinase inhibitor treatment. Our aim was to determine which carriers are responsible for bosutinib transport.

**Methods:**

K562S cells overexpressing the drug transporters ABCB1, ABCG2, and SLC22A1 were generated, characterized and used in proliferation assay and intracellular uptake and retention assay (IUR). In vivo experiments were performed in nude mice injected with K562S, K562DOX cells (overexpressing ABCB1), and K562DOX silenced for ABCB1 (K562DOX/sh P-GP).

**Results:**

The IUR assay using C-14 bosutinib showed that only ABCB1 was responsible for active bosutinib transport. K562DOX cells showed the lowest intracellular level of bosutinib, while K562DOX cells treated with the ABCB1 inhibitor verapamil showed intracellular bosutinib levels comparable with parental K562S. Proliferation assays demonstrated that K562DOX are resistant to bosutinib treatment while verapamil is able to restore the sensitivity to the drug. Nude mice injected with K562DOX and treated with bosutinib showed very limited response and quickly relapsed after stopping treatment while K562S as well as K562DOX/sh P-GP remained tumor-free.

**Conclusions:**

Our data suggest that the analysis of ABCB1 expression levels might help determine treatment options for patients exhibiting resistance to bosutinib.

**Electronic supplementary material:**

The online version of this article (doi:10.1186/s13045-015-0179-4) contains supplementary material, which is available to authorized users.

## Background

Since the discovery of imatinib, the treatment of chronic myeloid leukemia (CML) is based on tyrosine kinase inhibitors (TKIs) that selectively target BCR-ABL, the oncoprotein responsible for the disease. [1] Second-generation TKIs such as nilotinib and dasatinib have been recently approved as a first line treatment for CML [2, 3]. Bosutinib is a second-generation dual SRC/ABL TKI, approximately 10–30 times more potent than imatinib, and it was recently approved by FDA as a second line option for CML treatment [4].

Mutations in the kinase domain of BCR-ABL have been described as the main cause of resistance to all the TKIs during treatment, but they account for no more than 50 % of reported cases of resistance [5]. Among other resistance mechanisms possibly affecting drug efficacy, multidrug-resistance (MDR) transporters that reduce intracellular drug levels remain a continuing concern. So far, the following three transporter proteins have been identified as potential contributors to MDR development following imatinib treatment: the uptake drug transporter organic cation transporter 1 (OCT1, encoded by the *SLC22A1* gene) [6], the efflux drug transporter glycoprotein P (P-GP, MDR1, encoded by the *ABCB1* gene) [7], and the efflux drug transporter breast cancer resistance protein (BCRP, encoded by the *ABCG2* gene) [8]. Changes in the expression of the drug transporters (downmodulation of SLC22A1 or overexpression of ABCB1 and ABCG2) or single-nucleotide polymorphisms in these genes can cause imatinib resistance [9, 10].

Nilotinib and dasatinib are also subject to MDR mechanisms. While the efficacy of neither nilotinib nor dasatinib is affected by changes in SLC22A1 downmodulation [11, 12], nilotinib exhibits a concentration-dependent interaction with ABCB1 and ABCG2 [13, 14], and dasatinib is a substrate of both ABCB1 and ABCG2 [15, 16].

Given that bosutinib is a valuable option for the treatment of CML patients [4, 17], we examined whether SLC22A1, ABCB1, and ABCG2 are involved in its uptake and efflux with both in vitro assays and an in vivo model.

## Results

### Cell line characterization

**Fig. 1:**
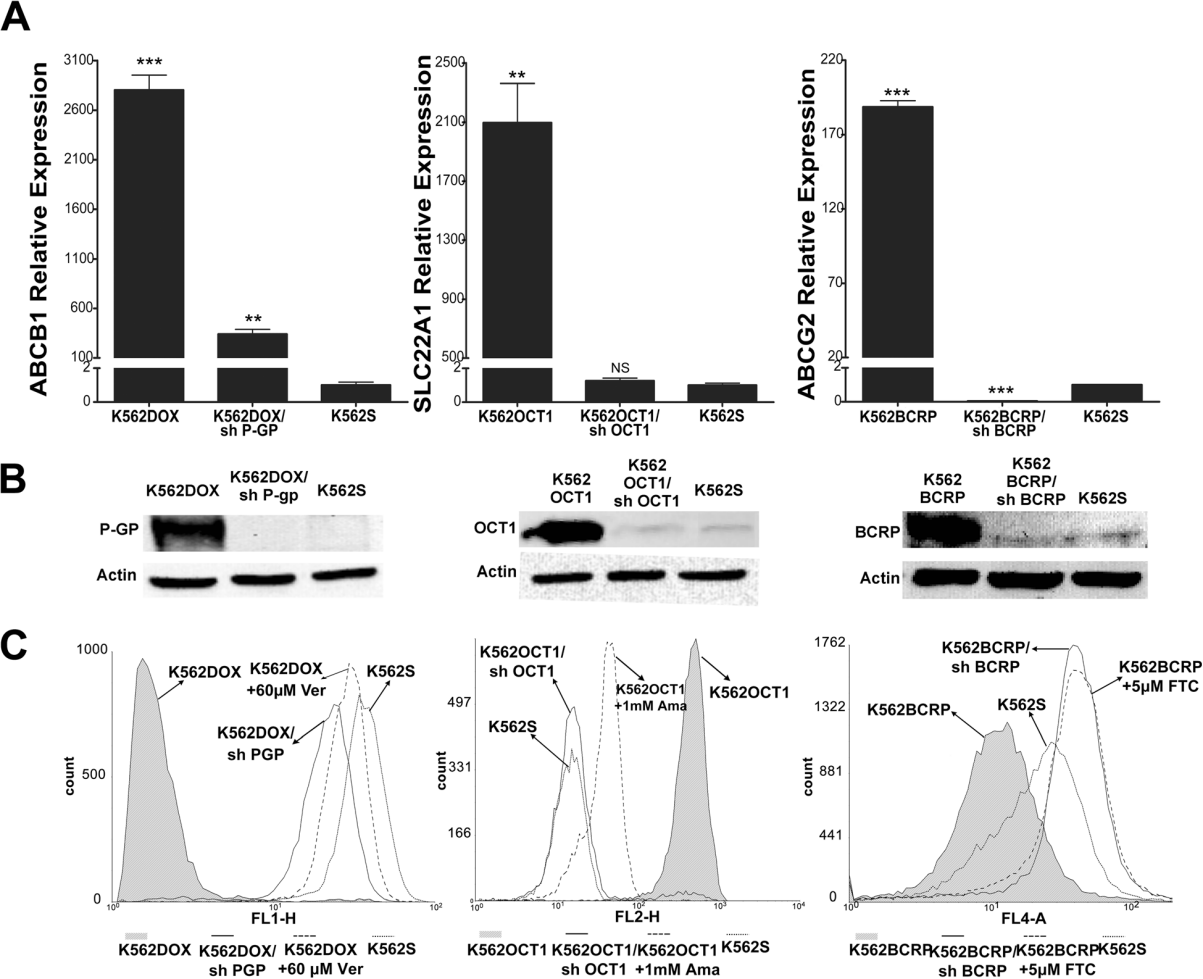
Evaluation of drug-transporter expression and functionality. **a** Evaluation of ABCB1, SLC22A1, and ABCG2 drug-transporter expression by real-time qPCR (RT-qPCR). Housekeeping GAPDH was used for intra-sample normalization. For each transporter, expression levels were normalized over the expression levels in K562S. Results are the average of three independent experiments ± SD. The statistical differences between expression levels of overexpressing or silenced cells and K562S cells were calculated with two-tailed unpaired student’s *t*-test, and a *p* value of 0.05 was chosen as the limit of statistical significance (** = *p* < 0.05 *** = *p* < 0.01). **b** Evaluation of drug-transporter expression levels by immunoblotting performed on whole cell lysate of overexpressing, silenced, and K562S cell lines. Specific antibodies were used for each drug transporter. Actin was used as a loading control. **c** Intracellular incorporation of known fluorescent substrates was evaluated by FACS analysis. On the *left panel*, rhodamine 123 incorporation for ABCB1 activity analysis, in the *middle panel*, 4-Di-2-ASP for SLC22A1 analysis, and in the *right panel*, pheophorbide A for ABCG2 analysis. In each graph, the *shaded area* corresponds to the overexpressing cell line, the *solid line* to the silenced cell line, the *dotted line* to the overexpressing cell lines pre-treated with drug-transporter inhibitor, and *shortly dashed line* to K562S

To investigate the involvement of ABCB1, ABCG2, and SLC22A1 in the active transport of bosutinib, we first characterized expression levels of functionally active drug transporters ABCB1, SLC22A1, and ABCG2 by means of RT-qPCR as well as immunoblotting analysis in the engineered cell lines described in the Materials and methods section. As expected, RT-qPCR analysis (Fig. 1a) showed an increased expression of ABCB1, SLC22A1, and ABCG2 in K562DOX, K562OCT1, and K562BCRP, respectively, in comparison to K562S parental cells. ABCB1 mRNA levels in K562DOX cells were approximately 2800-fold higher, SLC22A1 mRNA levels in K562OCT1 cells approximately 2100-fold, and ABCG2 mRNA levels in K562BCRP cells approximately 190-fold higher than corresponding mRNA levels observed in K562S parental cells. Stable silencing performed in the same overexpressing cell lines was able to successfully reduce the transcript levels of the different transporters (Fig. 1a). In particular, K562DOX/sh P-GP cells showed a 87.9 % decrease of ABCB1 levels, K562OCT1/sh OCT1 cells showed a 99.9 % decrease of SLC22A1 levels, and K562BCRP/sh BCRP cells showed a 99.9 % decrease of ABCG2 levels when compared to the corresponding overexpressing cells. Interestingly, K562BCRP/sh BCRP cells showed ABCG2 expression levels even lower than K562S, indicating that endogenous ABCG2 was also silenced.

Immunoblotting analysis confirmed the results obtained by RT-qPCR (Fig. 1b). The three cell lines overexpressing drug transporters showed a marked increase in protein expression compared with both silenced and K562S cell lines.

We subsequently checked the functionality of the transporters by measuring the intracellular incorporation of fluorescent substrates specific for each transporter. (Fig. 1c and Additional file 1: Figure S1). Overexpressing cells were either pre-treated or untreated with specific drug-transporter inhibitors. The cells overexpressing either ABCB1 or ABCG2 showed a decreased intracellular concentration of the fluorescent substrates rhodamine 123 (Rho 123) and pheophorbide A (PhA), respectively. As expected, K562S showed a high level of intracellular fluorescent substrates. In silenced cells as well as in cells treated with the specific drug-transporter inhibitors verapamil(Ver) or fumitremorgin C (FTC), fluorescence levels were similar to K562S. Consistent with the role of SLC22A1 in the uptake of the styrylpyridinium dye ASP, K562OCT1 cells showed higher ASP incorporation compared to K562S, while silencing of SLC22A1 or treatment with the specific SLC22A1 inhibitor amantadine (Ama) [18] led to decreased ASP incorporation levels similar to K562S. Taken together, our data confirm that these cell lines overexpress functionally active SLC22A1, ABCB1, or ABCG2.

### IUR assay for C-14 bosutinib cellular incorporation

**Fig. 2:**
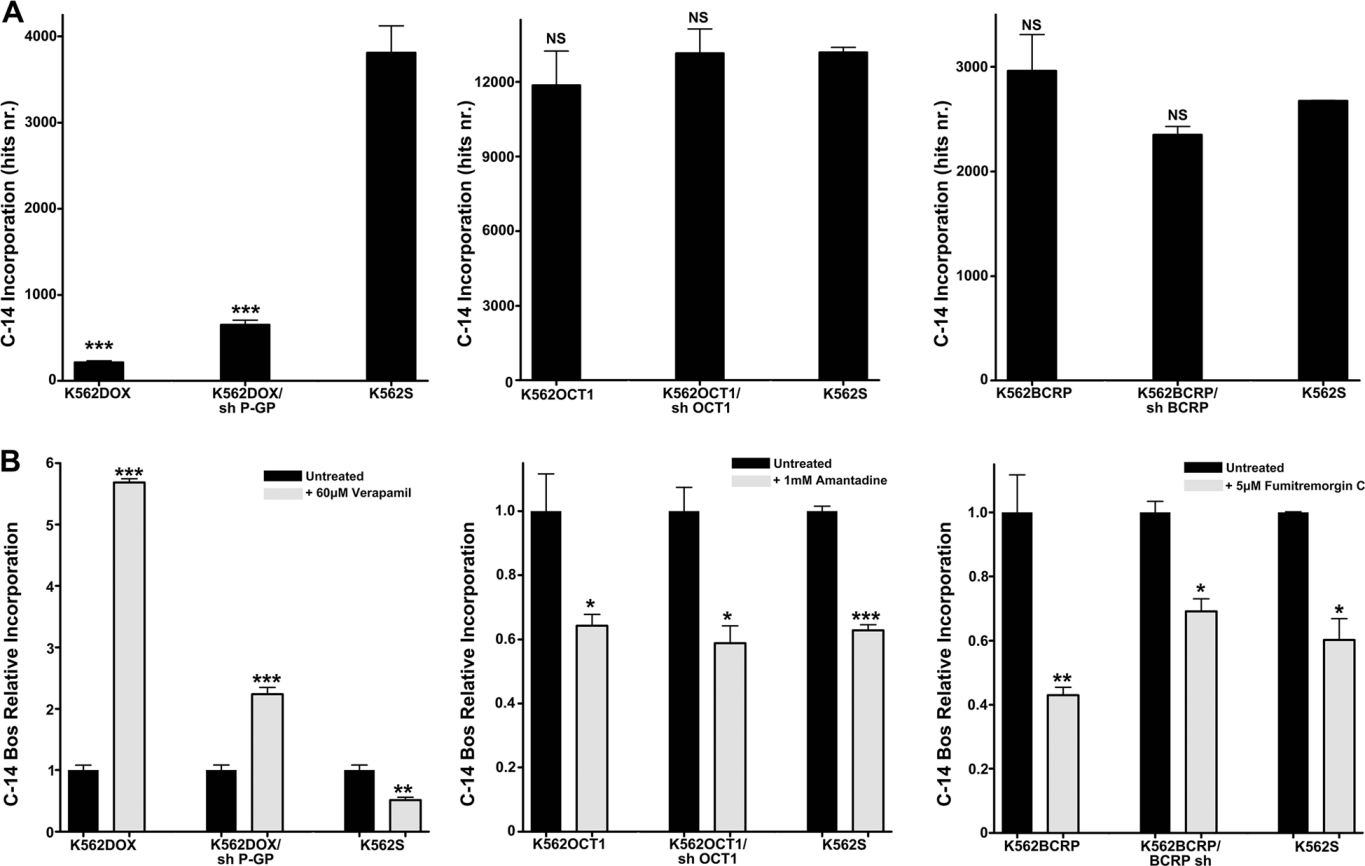
Intracellular uptake and retention assay. **a** Intracellular incorporation of C-14 bosutinib is reported as the number of hits obtained from beta-counter. The results derive from three independent experiments ± SD. For each drug transporter, the statistical difference between overexpressing or silenced cells and K562S was calculated with two-tailed unpaired student’s *t*-test, and a *p* value of 0.05 was chosen as the limit of statistical significance (** = *p* < 0.05 *** = *p* < 0.01 NS, not significant). **b** C-14 bosutinib intracellular accumulation after drug transporters inhibition. For each transporter, the cells were pre-treated with specific drug-transporter inhibitors. C-14 bosutinib incorporation obtained with the pre-treatment was normalized over C-14 bosutinib incorporation in the same cell line without the pre-treatment. Results are an average of three independent experiments. The statistical difference between untreated and pre-treated samples within each cell line was calculated with two-tailed unpaired student’s *t*-test, and a *p* value of 0.05 was chosen as the limit of statistical significance

The IUR assay has been extensively used to characterize the interaction of imatinib, nilotinib, and dasatinib with drug transporters [11, 15, 19, 20]. We used a similar approach to study the correlation between the expression of drug transporters and the intracellular concentration of C-14 bosutinib. While SLC22A1- and ABCG2-overexpressing cells did not show any marked difference in C-14 bosutinib incorporation, a statistically significant decrease was observed in K562DOX cells compared to K562S cells (Fig. 2a, *p* value 0.0003). K562DOX/sh P-GP cells exhibited an intermediate level of bosutinib incorporation, significantly decreased compared to K562S cell lines (Fig. 2a, *p* value 0.0006). These data suggest that ABCB1, but not SLC22A1 or ABCG2, regulates intracellular levels of bosutinib. We subsequently evaluated the effect of the specific transporter inhibitors verapamil, amantadine and fumitremorgin C, which inhibit ABCB1, SLC22A1, and ABCG2, respectively, on C-14 bosutinib incorporation (Fig. 2b). While SLC22A1 and ABCG2 overexpressing and silenced cells did not show significant differences in intracellular C-14 bosutinib levels when compared with K562S cells, K562DOX cells treated with verapamil showed a significant increase in intracellular C-14 bosutinib levels (5.7-fold; *p* value <0.0001) when compared to the corresponding untreated cells. K562DOX/sh P-GP exhibited a moderate increase (2.4-fold; *p* value 0.0008) in bosutinib levels. A modest decrease in bosutinib levels was observed in K562S cells (1.9 fold; *p* value 0.007) relative to cells not treated with verapamil, perhaps due to verapamil toxicity. Taken together, these data indicate that ABCB1 (but not SLC22A1 nor ABCG2) is involved in active bosutinib transport.

### K562DOX resistance to bosutinib and restoration of sensitivity by verapamil

**Fig. 3:**
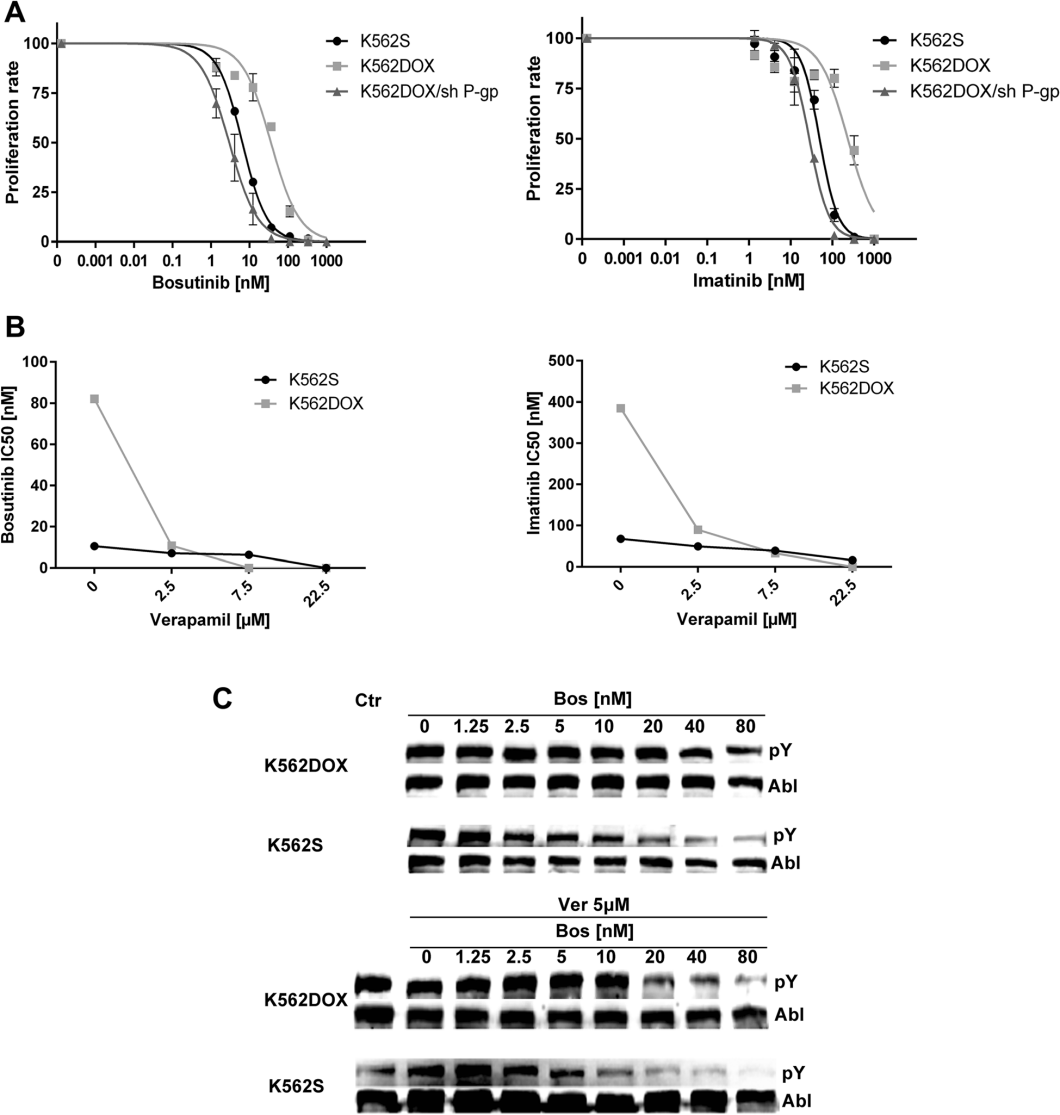
Effect of ABCB1 overexpression on bosutinib activity. **a** Proliferation assay on K562DOX, K562DOX/sh P-GP, and K562S. Cells were cultivated for 72 h in the presence of increasing concentration of either bosutinib or imatinib, and incorporation of tritiated thymidine was evaluated. Results presented are an average of at least three independent experiments. **b** Proliferation assay of K562DOX and K562S cells co-treated with verapamil and either bosutinib or imatinib. Verapamil was used at three selected concentrations, while bosutinib and imatinib were used within the same ranges used in Fig. 3a. The *y* axis reports means + SD of bosutinib or imatinib IC50s obtained. Data reported are a mean of three separate experiments. Non-linear regression was used to evaluate IC50s. **c** Evaluation of BCR-ABL phosphorylation levels in K562DOX and K562S cell lines, upon bosutinib treatment with or without verapamil co-treatment. Immunoblotting analysis was performed on whole cell lysates. ABL1 was used as a loading control

To assess the functional consequences of ABCB1 involvement in bosutinib efflux, we evaluated the anti-proliferative activity of bosutinib in K562DOX, K562DOX/sh P-GP, and K562S cells (Fig. 3a, Additional file 2: Figure S2a and Table 1). The IC50 value of bosutinib-treated cells is approximately 6–10 fold higher in K562DOX compared to either K562S or K562DOX/sh P-GP cells. Similar results were obtained with imatinib (IC50 fold change 5–8), as expected, since imatinib is a known ABCB1 substrate.

**Table 1:** IC50 values of bosutinib and imatinib calculated in the proliferation assay for K562S, K562DOX, and K562DOX/sh P-GP

	K562S	K562DOX	K562DOX/sh P-GP
Bosutinib	6.5 nM	36.1 nM	3 nM
Imatinib	49.3 nM	234.1 nM	28 nM

To test if the inhibition of ABCB1 correlates with a restoration of K562DOX sensitivity to imatinib or bosutinib treatment, we co-treated K562DOX and K562S cells with verapamil and either bosutinib or imatinib (Fig. 3b). As expected, the IC50s observed in K562DOX cells decreased significantly in presence of verapamil for both BCR-ABL inhibitors, even at the lowest concentration tested (*p* value <0.005). Conversely, the IC50s observed in K562S cells were not significantly affected by the presence of verapamil. These results indicate that ABCB1 overexpression decreases sensitivity to bosutinib, and inhibition of ABCB1 restores sensitivity to bosutinib in cells overexpressing ABCB1.

### The intracellular concentration of bosutinib correlates with BCR-ABL activity inhibition

Bosutinib, as well as the other TKIs, exerts a pro-apoptotic activity by directly reducing BCR-ABL kinase activity and autophosphorylation [21, 22]. To compare the effect of bosutinib treatment on BCR-ABL activity in K562DOX and K562S cells, we analyzed the phosphorylation levels of BCR-ABL at different drug concentrations (Fig. 3c and Additional file 2: Figure S2b). As expected, as bosutinib concentration increased, BCR-ABL phosphorylation levels decreased in K562S cells but not in K562DOX cells. However, when the bosutinib-treated cells were co-treated with the ABCB1 inhibitor verapamil, a decrease in the phosphorylation levels was also observed in K562DOX cells, while verapamil treatment alone had no effect on BCR-ABL phosphorylation. These results suggested that inhibition of ABCB1 transporter activity by verapamil caused intracellular bosutinib accumulation, resulting in the de-phosphorylation of BCR-ABL.

### In vivo effects of ABCB1 overexpression on bosutinib activity

**Fig. 4:**
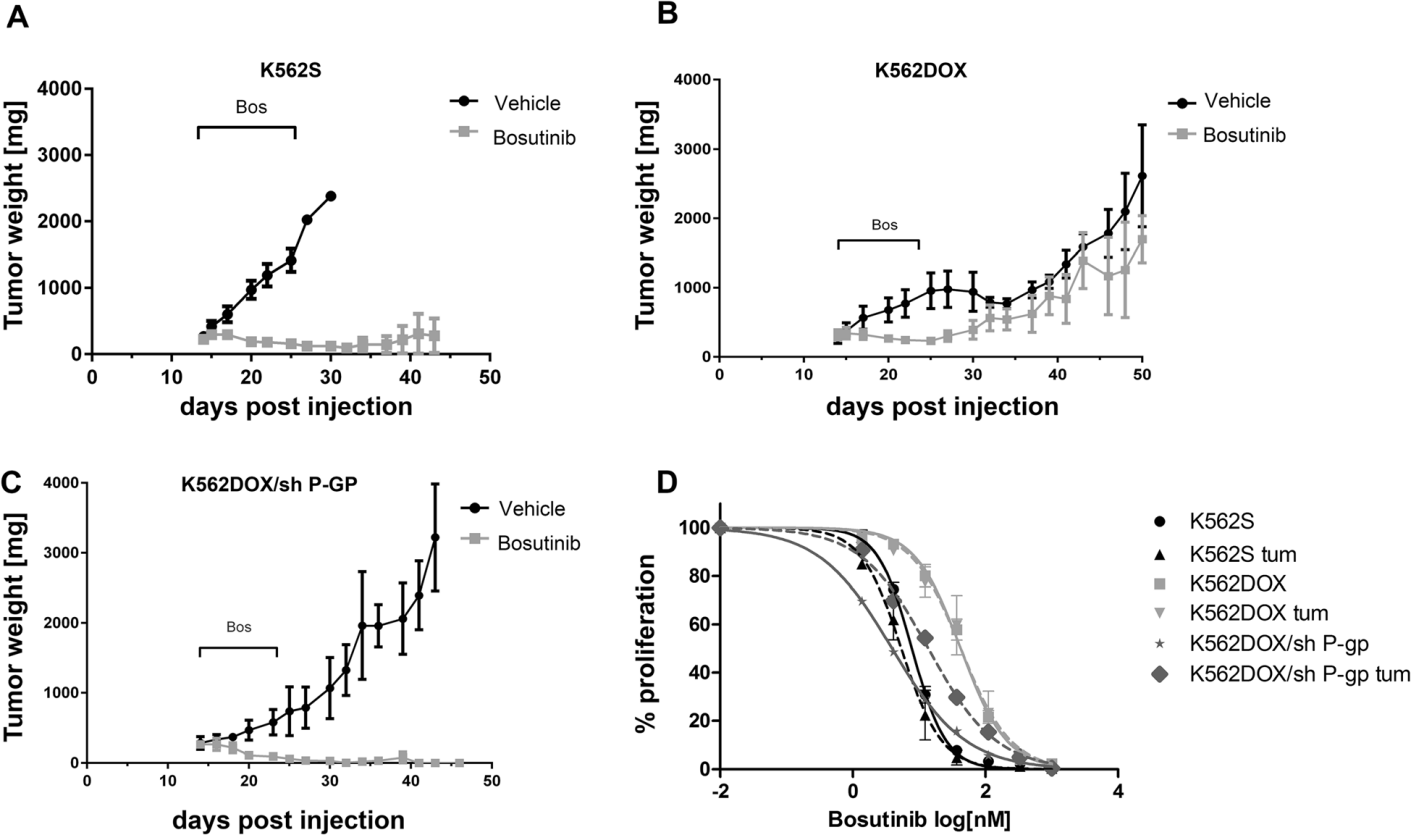
In vivo evaluation of ABCB1 overexpression effects on bosutinib activity. **a–c** Tumor-size measurements in mice injected with K562S (**a**), K562DOX (**b**), or K562DOX/sh P-GP (**c**). The treatment length with bosutinib (BOS) is indicated. **d** Proliferation experiment on ex vivo cells derived from K562S, K562DOX, and K562DOX/sh P-GP tumors. Cells were treated with increasing concentrations of bosutinib for 72 h. Results are the mean of two independent experiments. *p* values were calculated, comparing each cell line with the following corresponding tumor-derived cells: K562S versus K562S tum (*p* = 0.38), K562DOX versus K562DOXtum (*p* = 0.583), and K562DOX/sh P-GP versus K562DOX/sh P-GP tum (*p* = 0.003)

We next examined bosutinib anti-tumor activity as a function of ABCB1 overexpression in an in vivo xenograft model. A total of 39 mice were injected with either K562S (15 animals), K562DOX (14 animals), or K562DOX/sh P-GP (10 animals) cells. When tumors reached a weight of 200 mg (approximately day 14 after injection), mice in each group were randomized in two subgroups: one received bosutinib at 150 mg/kg, the other one vehicle alone, 5 days a week for 2 weeks. Tumor weight was measured for up to 1 month after treatment termination. As expected, bosutinib treatment caused tumor regression in mice with K562S. K562DOX mice exhibited an initial limited response to bosutinib treatment, but eventually relapsed after treatment was ended. In contrast, K562DOX/sh P-GP tumors in which ABCB1 expression was downregulated, showed complete sensitivity to bosutinib treatment, and regression was sustained over the period of observation (Fig. 4a–c).

Tumors were recovered from all the mice and analyzed for ABCB1 and BCR-ABL expression by RT-qPCR. ABCB1 analysis confirmed that cells derived from K562DOX tumors maintained high expression levels of the transporter when compared with the K562S tumors (Additional file 3: Figure S3). Tumor-derived cells were also used in the proliferation assay to evaluate the sensitivity to bosutinib treatment. IC50s observed in tumor-derived cells were fully comparable with those obtained using the corresponding cell lines, indicating that in vivo passage did not affect the behavior of these cells with respect to bosutinib treatment (Fig. 4d). To exclude the presence of Abl-kinase domain mutations as a source of bosutinib resistance, RNA was extracted from all the tumors, and the BCR-ABL kinase domain was amplified and sequenced. In all the cases, no mutations were found (Additional file 4: Supplementary material). Thus, the observed resistance to bosutinib treatment most likely resides in the elevated expression of ABCB1 leading to a reduced intracellular concentration of bosutinib.

## Discussion

The development of second-generation TKIs (dasatinib, nilotinib, bosutinib) was mainly directed toward overcoming resistance arising in patients treated with imatinib. Resistance to treatment is largely due to the presence of point mutations within the BCR-ABL kinase domain [23], although other mechanisms have been identified such as: BCR-ABL gene amplification [24] and duplication [25], clonal evolution due to the acquisition of other chromosomal abnormalities [26], low drug concentration in plasma [27, 28], alternative signaling pathway activation [29], epigenetic modifications [30], and MDR due to drug cellular uptake/efflux impairment [31]. Since many reports highlight the different transport patterns of nilotinib and dasatinib compared to imatinib, the two second-generation inhibitors were suggested as a valid treatment option to circumvent MDR to imatinib [11, 12]. To date, MDR affecting bosutinib has been investigated only by Hegedus and colleagues using in vitro assays [32].

In our work, we analyzed the interaction between bosutinib and the following three principal drug transporters involved in imatinib transport: ABCB1 [33], ABCG2 [8], or SLC22A1 [6]. Since the IUR assay has been extensively used to study multidrug resistance for imatinib, nilotinib, and dasatinib [11, 15, 34, 35], we used this assay to examine bosutinib transport. Our results demonstrated that intracellular levels of C-14 bosutinib were dependent on ABCB1 expression, with decreased intracellular bosutinib levels in cells with high ABCB1, and correspondingly increased intracellular bosutinib levels in cells with lower ABCB1 levels. In line with these findings, treatment with the ABCB1 inhibitor verapamil increased intracellular levels of bosutinib. In contrast, overexpressing either ABCG2 or SLC22A1 had no effect on bosutinib intracellular accumulation. Similarly, ABCG2 and SLC22A1 inhibition by fumitremorgin C and amantadine, respectively, did not result in any significant difference in intracellular bosutinib levels. These results suggest that in these cells, ABCG2 and SLC22A1 do not affect bosutinib transport, while ABCB1 catalyzes bosutinib efflux and is therefore a potential contributor to bosutinib resistance.

In line with these findings, the decreased intracellular levels of bosutinib in cells overexpressing ABCB1 are associated with a higher IC50, whereas treatment with verapamil reversed the effects of ABCB1 overexpression, but did not significantly affect the response to bosutinib of cells expressing native levels of ABCB1. As a known ABCB1 substrate, imatinib behaved similarly to bosutinib. ABCB1 overexpression also inhibited the ability of bosutinib to reduce BCR-ABL phosphorylation, whereas verapamil treatment restored bosutinib sensitivity. Thus, the observed resistance to bosutinib in the ABCB1 overexpressing cells in vitro is directly related to the increased levels of ABCB1.

ABCB1 overexpression also reduced the anti-tumor activity of bosutinib in vivo. Experiments on mice injected with control K562S cells, ABCB1 overexpressing K562DOX cells as well as K562DOX/sh P-GP, were performed to evaluate the possible effect of drug resistance on treatment response in vivo. As expected from the literature [36], K562S clearly responded to treatment, and the tumors remained not-measurable during the follow-up period. K562DOX cells initially responded partially to the treatment, but tumor growth eventually progressed in all mice. K562DOX/sh P-GP mice responded to the treatment, and no relapses were observed, confirming the role of ABCB1 overexpression in the resistance. Ex vivo analysis performed on tumor cells did not reveal any difference in BCR-ABL expression between K562S and K562DOX, excluding the possibility that the relapse might be due to an increased expression of the oncogenic protein. Moreover, we also excluded the presence of mutations as a possible cause of the resistance. Cells derived from K562DOX tumors showed ABCB1 levels comparable to the K562DOX cell line, indicating that they did not lose expression during in vivo experiments. IC50 values observed in the proliferation assay performed on ex vivo cells are similar with those obtained in vitro on cell lines not passaged through mice.

To our knowledge, this is the first demonstration of involvement of ABCB1 in bosutinib transport in both in vitro and in vivo settings. In previous works, Hegedus and colleagues [32], by combining several in vitro assays, concluded that bosutinib is neither a substrate of ABCB1 nor a substrate of ABCG2. Although our results agree in excluding a role of ABCG2 in bosutinib transport, there is a discrepancy with regard to the role of ABCB1. Such a difference could arise from the different assays used by the two groups as well as from the different concentration ranges tested. However, in vivo data included in the present work strongly support the premise that bosutinib is indeed a substrate of ABCB1 and that overexpression of this transporter is able to confer resistance to this drug.

In summary, we show here that intracellular concentrations of bosutinib are affected by the overexpression of the efflux transporter ABCB1. The reduced intracellular bosutinib concentration results in a reduction of its BCR-ABL inhibitory activity, thus leading to resistance. These data could be useful in the clinical management of Ph + leukemias. Further studies should be carried out in BCR-ABL^+^ patients under treatment with bosutinib to reveal any correlation between ABCB1 activity and clinical response which might allow the development of strategies to overcome bosutinib resistance.

## Materials and methods

### Chemical compounds and reagents

Bosutinib (SKI-606), and C-14 radiolabeled bosutinib (C-14 bosutinib) were obtained from Pfizer Inc, New York, NY. Both drugs were dissolved in DMSO to obtain a 10-mM stock solution. Imatinib (STI-571, Gleevec) was synthesized by Enrico Rosso, PhD, University of Venice, Italy and was dissolved in water to obtain a 10-mM stock solution.

Doxorubicin (Sigma-Aldrich, Saint Louis, MO) was dissolved in DMSO to obtain a final concentration of 10 mM. Geneticin and puromycin, (Euroclone Milan, Italy) were both dissolved in water to obtain stock solutions of 400 mg/mL and 25 mg/mL, respectively.

Verapamil hydrochloride (Sigma-Aldrich, Saint Louis, MO) was dissolved in ethanol to obtain a final concentration of 100 mM and used as a selective ABCB1 inhibitor. Amantadine hydrochloride (Sigma-Aldrich, Saint Louis, MO) was dissolved in ethanol to obtain a final concentration of 1 M and used as a selective SLC22A1 inhibitor. Fumitremorgin C (FTC) (Sigma-Aldrich, Saint Louis, MO) was dissolved in DMSO to obtain a final concentration of 1 mM and used as a selective ABCG2 inhibitor.

Rhodamine 123 (Rho-123) (Sigma-Aldrich, Saint Louis, MO), 4-Di-2-ASP (4-(4-Diethylaminostyryl)-1-methylpyridinium iodide, ASP) (Invitrogen, Carlsbad, CA), and Pheophorbide A (PhA) (Frontier Scientific, Logan, UT) were dissolved in DMSO to obtain a final concentration of 2 mM, 10 mM, and 1 mM, respectively.

### Cell lines and cell cultures

K562DOX (kind gift of JP Marie, Université Pierre et Marie Curie, Paris), K562DOX/sh P-GP (formerly K562DOX-siMDR/MM; kind gift of E. Gunsilius, Innsbruck Medical University, Austria), K562OCT1, K562OCT1/sh OCT1, K562BCRP, and K562BCRP/sh BCRP cell lines were derived from the leukemic BCR-ABL^+^ cell line K562S (K562 imatinib sensitive). Briefly, to obtain K562DOX cell line, K562S cells were treated with increasing concentrations of doxorubicin, allowing the selection of K562S cells expressing high levels of ABCB1 [37]. K562DOX/sh P-GP cell line was derived from K562DOX cells with a transposon-based vector-silencing system, allowing a stable ABCB1 downregulation [38]. K562OCT1 cell line was obtained by electroporation with pcDNA3.1 plasmid carrying a *SLC22A1* gene-coding sequence (kind gift of Prof. R. Clark and A. Giannoudis, Royal Liverpool University hospital, UK)[12]. K562BCRP cell line was obtained by electroporation using a pcDNA3.1 plasmid carrying a *ABCG2* gene-coding sequence isolated from the carrier plasmid pCMV6-XL5 obtained from Origene (Rockville, MD). Stable SLC22A1 and ABCG2 silencing were obtained after stable transfections with MISSION shRNA plasmid DNA (Sigma-Aldrich, Sant Louis, MO) in K562OCT1 and K562BCRP cell lines, respectively. All cell lines were grown in standard conditions with RPMI 1640 medium (Lonza Cambrex, East Rutherford, NJ) supplemented with 10 % fetal bovine serum (FBS) (Euroclone, Milan, Italy), 2 mM L glutamine, 100-units/mL penicillin G, 80-μg/mL gentamicin, and 20-mM hepes. Cell lines were kept in selective pressure with the following reagents: 1-μM doxorubicin for K562DOX, 1-μM doxorubicin, and 1-mg/mL geneticin for K562DOX/sh P-GP, 1-mg/mL geneticin for K562OCT1 and K562BCRP, 1-mg/mL geneticin, and 1-μM Puromycin for K562OCT1/sh OCT1 and K562BCRP/sh BCRP.

### Reverse transcription and qPCR

RNA extraction from all the cell lines was performed with Trizol reagent (Invitrogen, Carlsbad, CA), according to the manufacturer’s protocol. After quantification, RNA was reverse transcribed to obtain cDNA with TaqMan kit (Applied Biosystems, Foster City, CA), according to the standard protocol. Real-time qPCR (RT-qPCR) was performed to assess the transcription levels of ABCB1, SLC22A1, and ABCG2 in all the cell lines. The following Sybr-Green primers were used for the analysis: *ABCB1* for: 5’- TGGAGGAAGACATGACCAGG-3’; ABCB1 rev: 5’-CAAGACCTCTTCAGCTACTGC-3’; *SLC22A1* for: 5’-GGGCAGCCTGCCTCGTCATG-3’; SLC22A1 rev: 5’-ACCTCCCTCAGCCTGAAGAC-3’; *ABCG2* for: 5’-TTAGGATTGAAGCCAAAGG-3’; ABCG2 rev: 5’-TAGGCAATTGTGAGGAAAATA-3’. The housekeeping gene *GAPDH* was used for intra-sample normalization: GAPDH for: 5’-TGCACCACCAACTGCTTAGC-3’; GAPDH rev: 5’-GGCATGGACTGTGGTCATGAG-3’.

### Immunoblotting

For the evaluation of the expression levels of the drug transporters, 10^7^ cells were lysed with a specific protocol for membrane-protein purification. Briefly, K562DOX, K562DOX/sh P-GP, K562OCT1, K562OCT1/sh OCT1, and K562S were lysed adding boiling 125-mM Tris–HCl pH 6.8, SDS 2 % solution supplemented with protease inhibitors. After resuspension and further boiling, samples were sonicated and Laemmli buffer was added. K562BCRP, K562BCRP/sh BCRP, and K562S were lysed using a hypotonic lysis buffer, 100 mM KCl, 2 mM MgCl1, 100 mM Tris–HCl (pH 7.4), 1 % SDS supplemented with protease inhibitors. After sonication, Laemmli buffer was added and lysates were heated at 60 °C for 1 h.

Samples were loaded on SDS-PAGE, transferred to nitrocellulose, and probed with the following different antibodies: monoclonal ABCB1 antibody C494 (Abcam, Cambridge UK); polyclonal SLC22A1 antibody AB1 (Sigma-Aldrich, Saint Louis MO) monoclonal ABCG2 antibody BXP-21 (Enzo Life Sciences, AG, Lausen, Switzerland), and polyclonal actin antibody (Sigma-Aldrich, Saint Louis MO.

For the evaluation of phosphorylation levels of BCR-ABL, K562DOX, and K562S, cells were treated with serial dilutions of bosutinib for 8 h at standard conditions. Eventually, a pre-treatment of 1 h with 5-μM verapamil was performed to inhibit the ABCB1 activity. Ten million cells were lysed using a standard protocol described previously [21]. Samples were loaded on SDS-PAGE, transferred to nitrocellulose, and probed with anti-phosphotyrosine antibody (clone 4G10, Millipore, Billerica MA). BCR-ABL levels were probed with anti-c-ABL (K12 clone, Santa Cruz Biotechnology, Santa Cruz CA).

### FACS analysis

Functional activity of ABCB1, SLC22A1, and ABCG2 was evaluated measuring the intracellular accumulation of known transporter substrates such as: Rho 123 for ABCB1, ASP for SLC22A1, and Ph A for ABCG2, in the presence or absence of the selective transporter inhibitors. One million cells were incubated for 30 min with fluorescence substrates and, where indicated, a 2-h pre-treatment with drug-transporter inhibitors was also performed. Cells were washed twice with ice-cold PBS before FACS analysis. Rho 123 and ASP incorporation was determined with the BD FACSort (Becton Dickinson, San Jose CA); Ph A incorporation was determined with the BD FACScantoI (Becton Dickinson, San Jose CA).

### Intracellular uptake and retention assay (IUR)

Incorporation of C-14 radiolabeled bosutinib (C-14 bosutinib) was evaluated in all the cell lines in the presence or absence of specific drug-transporter inhibitors. One million cells were resuspended in 2 mL of complete medium. Drug-transporter inhibitors were added at selected concentrations (described above) for 2 h. C-14 bosutinib 1 μM was added for an additional hour of incubation. Cells were washed thrice with 1-mL ice-cold PBS, resuspended in 30-μL PBS, and spotted on a membrane filter. Radioactivity levels were detected on a 1450 MicroBeta Trilux β-counter (Perkin Elmer, Waltham MA).

### Proliferation and co-treatment assay

K562DOX, K562DOX/sh P-GP, and K562S cells were seeded in a 96-well plate at a concentration of 10^4^ cells/well. Imatinib and bosutinib were added at increasing concentrations. For the co-treatment assay, imatinib and bosutinib were used at serial concentrations, while the ABCB1 inhibitor verapamil was added at four different concentrations below its IC50. For both assays, tritiated thymidine was added after 72 h; cells were then harvested, and the levels of tritiated thymidine were evaluated by a 1450 MicroBeta Trilux β-counter (Perkin Elmer, Waltham MA).

### In vivo experiments

Six- to eight-week-old female athymic nu/nu mice were purchased from Harlan Laboratories (San Pietro al Natisone-Udine, Italy) and kept under standard laboratory conditions, according to the guidelines of the University of Milano-Bicocca (Unimib, Monza, Italy). The study was approved by the Italian Ministry of Public Health (authorization 176/2009-B). K562S, K562DOX, and K562DOX/sh P-GP cells were resuspended at a concentration of 10^8^/ml in a suspension 1:1 PBS-Matrigel (BD biosciences- Franklin Lakes, NJ USA), and 0.2 ml were injected sub cutaneously on the right flank. Tumor weight was calculated by the formula tumor weight (mg) = (d^2^ × D/2), where d and D are the shortest and longest diameters of the tumor, respectively, measured in millimeters. When tumors average reached 200 mg, treatment started. Mice received 150-mg/kg bosutinib by oral gavage, and placebo-treated animals received vehicle alone (0.5 % methylcellulose-0.4 % Tween 80). Mice were treated once a day for 5 days/week for 2 weeks [21]. After sacrifice, tumors were collected, and recovered cells were used to perform further analysis.

### Statistical analysis

All the statistical analysis, data, and graph elaborations were run on GraphPad software analysis program (Prism, San Diego, CA).

## Additional files

Additional file 1: **Supplementary Figure S1.** Confocal microscopy analysis of ABCB1 activity. K562S and K562DOX cells were treated as previously described for FACS analysis (Fig. 1), and an equal amount of cells were acquired using a Zeiss LSM 710 confocal laser-scanning microscope (Jena, Germany). Samples were acquired both in contrast phase and using specific settings for rhodamine excitation and emission (Laser 561 nm and an emission window between 570 and 640 nm) using a ×20 air-phase or ×40 oil-phase objective applying also an additional hardware zoom to better define the fluorescent subcellular localization. The acquisition parameters were set on the signal deriving from the K562S samples and kept constant for all the other samples. Phase contrast images were acquired in order to assess the presence of a comparable morphology and cell number in all the analyzed samples.

Additional file 2: **Supplementary Figure S2.** A) IC50 values of imatinib and bosutinib. IC50 values (mean ± SD) of imatinib and bosutinib calculated from non-linear regression-proliferation curves reported in Fig. 3a. The statistical differences were calculated with two-tailed unpaired student’s *t*-test, and a *p* value of 0.05 was chosen as the limit of statistical significance. B) Densitometric analysis. Western blot (Fig. 3c) was analyzed by densitometry normalizing the anti-phosphotyrosine signal over its loading control (anti-abl). Relative signal intensity in the control lane was set as 100 %.

Additional file 3: **Supplementary Figure S3.** Evaluation of ABCB1 expression by real-time qPCR in cells recovered from tumor and the corresponding cell line. Housekeeping GAPDH was used for intra-sample normalization. Expression levels were normalized over the levels in K562S. Results are the average of three independent experiments ± SD. The statistical data were calculated with two-tailed unpaired student’s *t*-test, and a *p* value of 0.05 was chosen as the limit of statistical significance.

Additional file 4: **Supplementary material.** Sanger sequencing analysis of the Abl kinase domain in tumor derived cells.
