# Supplementary figures and images for: In vitro and in vivo identification of ABCB1 as an efflux transporter of bosutinib
Source: J Hematol Oncol. 2015 Jul 7;8:81. doi: 10.1186/s13045-015-0179-4 (PMC4491863; doi:10.1186/s13045-015-0179-4)

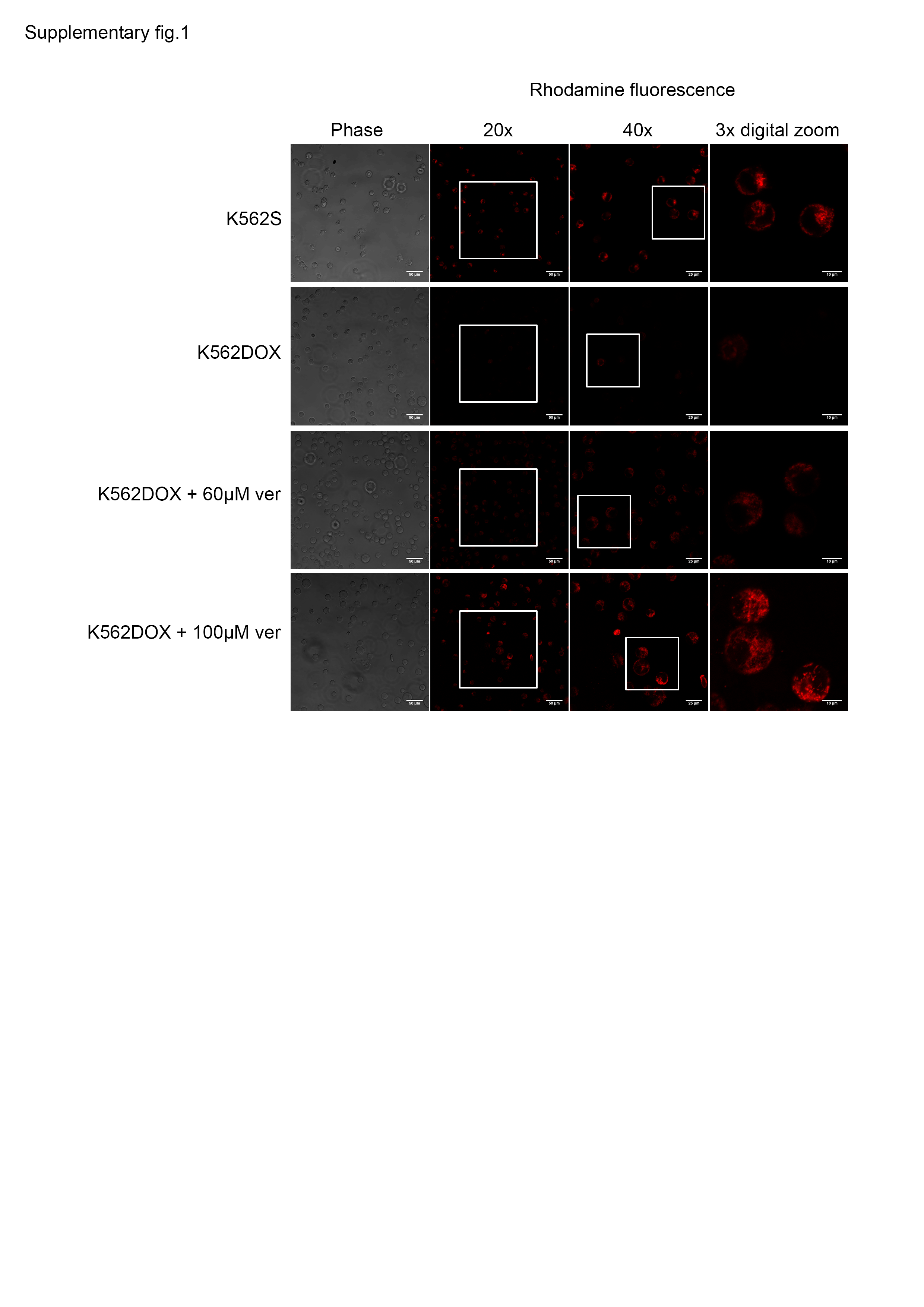

Supplement: Supplementary file 1 — Supplementary Figure S1. Confocal microscopy analysis of ABCB1 activity. K562S and K562DOX cells were treated as previously described for FACS analysis (Fig. 1), and an equal amount of cells were acquired using a Zeiss LSM 710 confocal laser-scanning microscope (Jena, Germany). Samples were acquired both in contrast phase and using specific settings for rhodamine excitation and emission (Laser 561 nm and an emission window between 570 and 640 nm) using a ×20 air-phase or ×40 oil-phase objective applying also an additional hardware zoom to better define the fluorescent subcellular localization. The acquisition parameters were set on the signal deriving from the K562S samples and kept constant for all the other samples. Phase contrast images were acquired in order to assess the presence of a comparable morphology and cell number in all the analyzed samples. [file 13045_2015_179_MOESM1_ESM.tif]

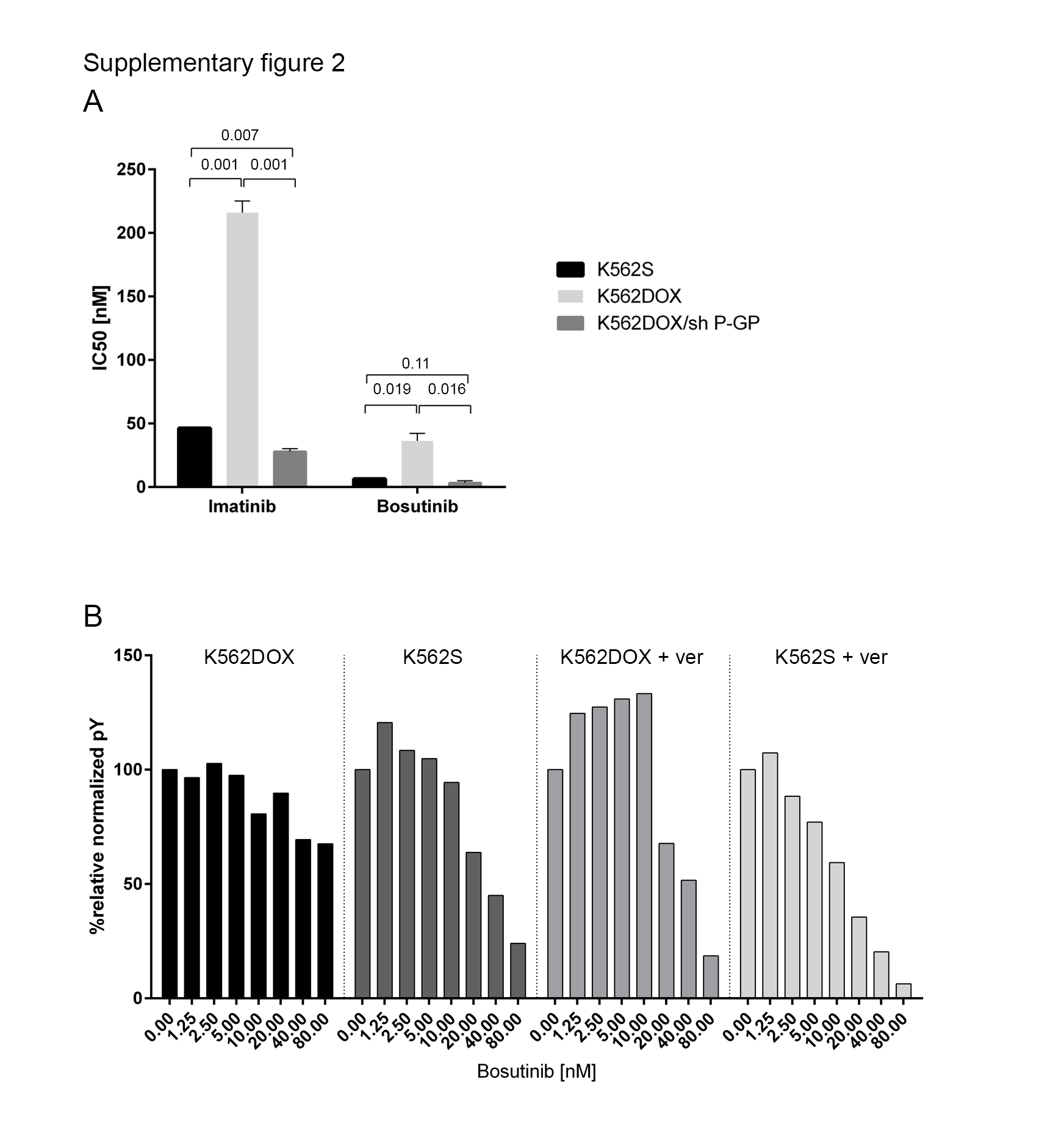

Supplement: Supplementary file 2 — Supplementary Figure S2. A) IC50 values of imatinib and bosutinib. IC50 values (mean ± SD) of imatinib and bosutinib calculated from non-linear regression-proliferation curves reported in Fig. 3a. The statistical differences were calculated with two-tailed unpaired student’s t-test, and a p value of 0.05 was chosen as the limit of statistical significance. B) Densitometric analysis. Western blot (Fig. 3c) was analyzed by densitometry normalizing the anti-phosphotyrosine signal over its loading control (anti-abl). Relative signal intensity in the control lane was set as 100 %. [file 13045_2015_179_MOESM2_ESM.tif]

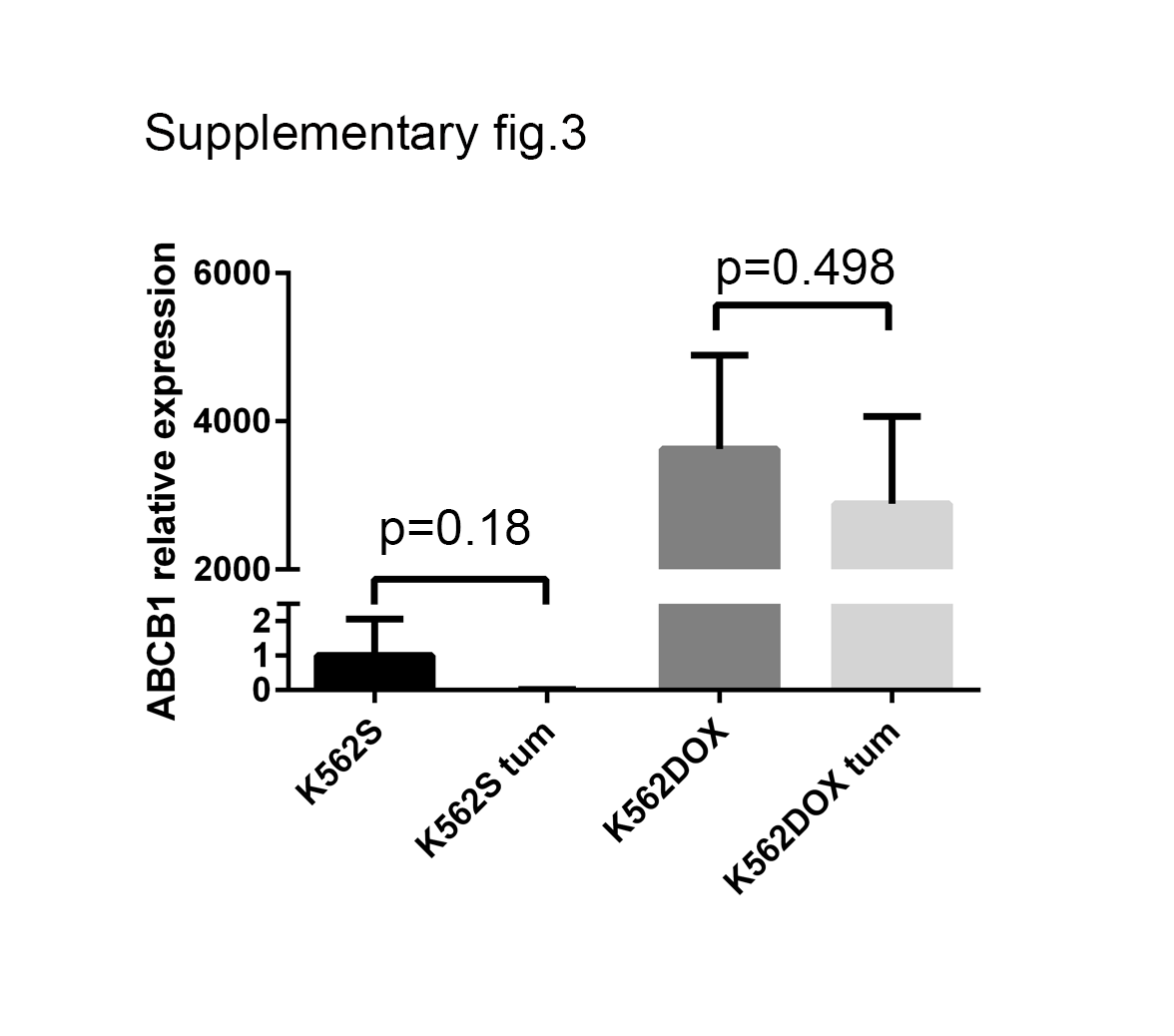

Supplement: Supplementary file 3 — Supplementary Figure S3. Evaluation of ABCB1 expression by real-time qPCR in cells recovered from tumor and the corresponding cell line. Housekeeping GAPDH was used for intra-sample normalization. Expression levels were normalized over the levels in K562S. Results are the average of three independent experiments ± SD. The statistical data were calculated with two-tailed unpaired student’s t-test, and a p value of 0.05 was chosen as the limit of statistical significance. [file 13045_2015_179_MOESM3_ESM.tif]
