# Supplementary material for: In vitro and in vivo identification of ABCB1 as an efflux transporter of bosutinib
Source: J Hematol Oncol. 2015 Jul 7;8:81. doi: 10.1186/s13045-015-0179-4 (PMC4491863; doi:10.1186/s13045-015-0179-4)
Supplement: Supplementary file 4 — Supplementary material. Sanger sequencing analysis of the Abl kinase domain in tumor derived cells. [file 13045_2015_179_MOESM4_ESM.docx]

**Supplementary material**

**Sequences from tumor samples**.

RNA was extracted from tumor samples, retrotranscribed and amplified using Titan OneTube PCR (Roche Diagnostic) accordingly to manufacturer instruction with the following primers: Bcrb2c_for(5’-CAGATGCTGACCAACTCGTGT-3’) and Abl_7rev(5’-CCGGAAGGAGCTGCTGCGTT-3’) in order to amplify the kinase domain of Bcr-Abl. Sanger sequence was performed using Abl_4rev primer (5’- CTTCTCTAGCAGCTCATACAC-3’). Three representative sequences aligned against the cABL refseq are shown. In all the cases no mutations within the kinase domain (aa 210-440, underligned) were observed.

NT 587 636

cAbl (580) ATCAATGGCAGCTTCTTGGTGCGTGAGAGTGAGAGCAGTCCTGGCCAGAG

K562DOXtum_Bos2 rc (206) ATCAATGGCAGCTTCTTGGTGCGTGAGAGTGAGAGCAGTCCTGGCCAGAG

K562DOXtum_Bos1 rc (225) ATCAATGGCAGCTTCTTGGTGCGTGAGAGTGAGAGCAGTCCTGGCCAGAG

K562DOXtum_Bos3 rc (303) ATCAATGGCAGCTTCTTGGTGCGTGAGAGTGAGAGCAGTCCTGGCCAGAG

637 686

cAbl (630) GTCCATCTCGCTGAGATACGAAGGGAGGGTGTACCATTACAGGATCAACA

K562DOXtum_Bos2 rc (256) GTCCATCTCGCTGAGATACGAAGGGAGGGTGTACCATTACAGGATCAACA

K562DOXtum_Bos1 rc (275) GTCCATCTCGCTGAGATACGAAGGGAGGGTGTACCATTACAGGATCAACA

K562DOXtum_Bos3 rc (353) GTCCATCTCGCTGAGATACGAAGGGAGGGTGTACCATTACAGGATCAACA

687 736

cAbl (680) CTGCTTCTGATGGCAAGCTCTACGTCTCCTCCGAGAGCCGCTTCAACACC

K562DOXtum_Bos2 rc (306) CTGCTTCTGATGGCAAGCTCTACGTCTCCTCCGAGAGCCGCTTCAACACC

K562DOXtum_Bos1 rc (325) CTGCTTCTGATGGCAAGCTCTACGTCTCCTCCGAGAGCCGCTTCAACACC

K562DOXtum_Bos3 rc (403) CTGCTTCTGATGGCAAGCTCTACGTCTCCTCCGAGAGCCGCTTCAACACC

737 786

cAbl (730) CTGGCCGAGTTGGTTCATCATCATTCAACGGTGGCCGACGGGCTC**ATCAC**

K562DOXtum_Bos2 rc (356) CTGGCCGAGTTGGTTCATCATCATTCAACGGTGGCCGACGGGCTCATCAC

K562DOXtum_Bos1 rc (375) CTGGCCGAGTTGGTTCATCATCATTCAACGGTGGCCGACGGGCTCATCAC

K562DOXtum_Bos3 rc (453) CTGGCCGAGTTGGTTCATCATCATTCAACGGTGGCCGACGGGCTCATCAC

787 836

cAbl (780) **CACGCTCCATTATCCAGCCCCAAAGCGCAACAAGCCCACTGTCTATGGTG**

K562DOXtum_Bos2 rc (406) CACGCTCCATTATCCAGCCCCAAAGCGCAACAAGCCCACTGTCTATGGTG

K562DOXtum_Bos1 rc (425) CACGCTCCATTATCCAGCCCCAAAGCGCAACAAGCCCACTGTCTATGGTG

K562DOXtum_Bos3 rc (503) CACGCTCCATTATCCAGCCCCAAAGCGCAACAAGCCCACTGTCTATGGTG

837 886

cAbl (830) **TGTCCCCCAACTACGACAAGTGGGAGATGGAACGCACGGACATCACCATG**

K562DOXtum_Bos2 rc (456) TGTCCCCCAACTACGACAAGTGGGAGATGGAACGCACGGACATCACCATG

K562DOXtum_Bos1 rc (475) TGTCCCCCAACTACGACAAGTGGGAGATGGAACGCACGGACATCACCATG

K562DOXtum_Bos3 rc (553) TGTCCCCCAACTACGACAAGTGGGAGATGGAACGCACGGACATCACCATG

887 936

cAbl (880) **AAGCACAAGCTGGGCGGGGGCCAGTACGGGGAGGTGTACGAGGGCGTGTG**

K562DOXtum_Bos2 rc (506) AAGCACAAGCTGGGCGGGGGCCAGTACGGGGAGGTGTACGAGGGCGTGTG

K562DOXtum_Bos1 rc (525) AAGCACAAGCTGGGCGGGGGCCAGTACGGGGAGGTGTACGAGGGCGTGTG

K562DOXtum_Bos3 rc (603) AAGCACAAGCTGGGCGGGGGCCAGTACGGGGAGGTGTACGAGGGCGTGTG

937 986

cAbl (930) **GAAGAAATACAGCCTGACGGTGGCCGTGAAGACCTTGAAGGAGGACACCA**

K562DOXtum_Bos2 rc (556) GAAGAAATACAGCCTGACGGTGGCCGTGAAGACCTTGAAGGAGGACACCA

K562DOXtum_Bos1 rc (575) GAAGAAATACAGCCTGACGGTGGCCGTGAAGACCTTGAAGGAGGACACCA

K562DOXtum_Bos3 rc (653) GAAGAAATACAGCCTGACGGTGGCCGTGAAGACCTTGAAGGAGGACACCA

987 1036

cAbl (980) **TGGAGGTGGAAGAGTTCTTGAAAGAAGCTGCAGTCATGAAAGAGATCAAA**

K562DOXtum_Bos2 rc (606) TGGAGGTGGAAGAGTTCTTGAAAGAAGCTGCAGTCATGAAAGAGATCAAA

K562DOXtum_Bos1 rc (625) TGGAGGTGGAAGAGTTCTTGAAAGAAGCTGCAGTCATGAAAGAGATCAAA

K562DOXtum_Bos3 rc (703) TGGAGGTGGAAGAGTTCTTGAAAGAAGCTGCAGTCATGAAAGAGATCAAA

1037 1086

cAbl (1030) **CACCCTAACCTGGTGCAGCTCCTTGGGGTCTGCACCCGGGAGCCCCCGTT**

K562DOXtum_Bos2 rc (656) CACCCTAACCTGGTGCAGCTCCTTGGGGTCTGCACCCGGGAGCCCCCGTT

K562DOXtum_Bos1 rc (675) CACCCTAACCTGGTGCAGCTCCTTGGGGTCTGCACCCGGGAGCCCCCGTT

K562DOXtum_Bos3 rc (753) CACCCTAACCTGGTGCAGCTCCTTGGGGTCTGCACCCGGGAGCCCCCGTT

1087 1136

cAbl (1080) **CTATATCATCACTGAGTTCATGACCTACGGGAACCTCCTGGACTACCTGA**

K562DOXtum_Bos2 rc (706) CTATATCATCACTGAGTTCATGACCTACGGGAACCTCCTGGACTACCTGA

K562DOXtum_Bos1 rc (725) CTATATCATCACTGAGTTCATGACCTACGGGAACCTCCTGGACTACCTGA

K562DOXtum_Bos3 rc (803) CTATATCATCACTGAGTTCATGACCTACGGGAACCTCCTGGACTACCTGA

1137 1186

cAbl (1130) **GGGAGTGCAACCGGCAGGAGGTGAACGCCGTGGTGCTGCTGTACATGGCC**

K562DOXtum_Bos2 rc (756) GGGAGTGCAACCGGCAGGAGGTGAACGCCGTGGTGCTGCTGTACATGGCC

K562DOXtum_Bos1 rc (775) GGGAGTGCAACCGGCAGGAGGTGAACGCCGTGGTGCTGCTGTACATGGCC

K562DOXtum_Bos3 rc (853) GGGAGTGCAACCGGCAGGAGGTGAACGCCGTGGTGCTGCTGTACATGGCC

1187 1236

cAbl (1180) **ACTCAGATCTCGTCAGCCATGGAGTACCTGGAGAAGAAAAACTTCATCCA**

K562DOXtum_Bos2 rc (806) ACTCAGATCTCGTCAGCCATGGAGTACCTGGAGAAGAAAAACTTCATCCA

K562DOXtum_Bos1 rc (825) ACTCAGATCTCGTCAGCCATGGAGTACCTGGAGAAGAAAAACTTCATCCA

K562DOXtum_Bos3 rc (903) ACTCAGATCTCGTCAGCCATGGAGTACCTGGAGAAGAAAAACTTCATCCA

1237 1286

cAbl (1230) **CAGAGATCTTGCTGCCCGAAACTGCCTGGTAGGGGAGAACCACTTGGTGA**

K562DOXtum_Bos2 rc (856) CAGAGATCTTGCTGCCCGAAACTGCCTGGTAGGGGAGAACCACTTGGTGA

K562DOXtum_Bos1 rc (875) CAGAGATCTTGCTGCCCGAAACTGCCTGGTAGGGGAGAACCACTTGGTGA

K562DOXtum_Bos3 rc (953) CAGAGATCTTGCTGCCCGAAACTGCCTGGTAGGGGAGAACCACTTGGTGA

1287 1336

cAbl (1280) **AGGTAGCTGATTTTGGCCTGAGCAGGTTGATGACAGGGGACACCTACACA**

K562DOXtum_Bos2 rc (906) AGGTAGCTGATTTTGGCCTGAGCAGGTTGATGACAGGGGACACCTACACA

K562DOXtum_Bos1 rc (925) AGGTAGCTGATTTTGGCCTGAGCAGGTTGATGACAGGGGACACCTACACA

K562DOXtum_Bos3 rc (1003) AGGTAGCTGATTTTGGCCTGAGCAGGTTGATGACAGGGGACACCTACACA

1337 1386

cAbl (1330) **GCCCATGCTGGAGCCAAGTTCCCCATCAAATGGACTGCACCCGAGAGCCT**

K562DOXtum_Bos2 rc (956) GCCCATGCTGGAGCCAAGTTCCCCATCAAATGGACTGCACCCGAGAGCCT

K562DOXtum_Bos1 rc (975) GCCCATGCTGGAGCCAAGTTCCCCATCAAATGGACTGCACCCGAGAGCCT

K562DOXtum_Bos3 rc (1053) GCCCATGCTGGAGCCAAGTTCCCCATCAAATGGACTGCACCCGAGAGCCT

1387 1436

cAbl (1380) **GGCCTACAACAAGTTCTCCATCAAGTCCGACGTCTGGGCATTTGGAGTAT**

K562DOXtum_Bos2 rc (1006) GGCCTACAACAAGTTCTCCATCAAGTCCGACGTCTGGGCATTTGGAGTAT

K562DOXtum_Bos1 rc (1025) GGCCTACAACAAGTTCTCCATCAAGTCCGACGTCTGGGCATTTGGAGTAT

K562DOXtum_Bos3 rc (1103) GGCCTACAACAAGTTCTCCATCAAGTCCGACGTCTGGGCATTTGGAGTAT

1437 1478

cAbl (1430) **TGCTTTGGGAAATTGCTACCTATGGCATGTCCCCTTACCCGG**

K562DOXtum_Bos2 rc (1056) TGCTTTGGGAAATTGCTACCTATGGCATGTCCCGT-ACCCGG

K562DOXtum_Bos1 rc (1075) TGCTTTGGGAAATTGCTACCTATGGCATGTCCCCT-ACCCGG

K562DOXtum_Bos3 rc (1153) TGCTTTGGGAAATTGCTACCTATGGCATT---CCT-ACCCGG

**Supplementary figures legends**

**Supplementary figure 1:**

**Confocal microscopy analysis of ABCB1 activity**. K562S and K562DOX cells were treated as previously described for FACS analysis (figure 1) and an equal amount of cells was acquired using a Zeiss LSM 710 confocal laser-scanning microscope (Jena, Germany). Samples were acquired both in contrast phase and using specific settings for rhodamine excitation and emission (Laser 561nm and an emission window between 570nm and 640nm) using a 20x air-phase or 40x oil-phase objective applying also an additional hardware zoom to better define the fluorescent subcellular localization. The acquisition parameters were set on the signal deriving from the K562S samples and kept constant for all the other samples. Phase contrast images were acquired in order to assess the presence of a comparable morphology and cell number in all the analyzed samples.

**Supplementary figure 2:**

**A)IC50 values of imatinib and bosutinib.** IC50 values (mean ± SD) of imatinib and bosutinib calculated from non-linear regression proliferation curves reported in figure 3A. The statistical differences were calculated with two-tailed unpaired t-student’s t-test, and p-value of 0.05 was chosen as the limit of statistical significance. **B) Densitometric analysis.** Western blot (figure 3C) was analyzed by densitometry normalizing the anti-phosphotyrosine signal over its loading control (anti-abl). Relative signal intensity in the control lane was set as 100%.

**Supplementary figure 3:**

**Evaluation of ABCB1 expression by Real time qPCR in cells recovered from tumor and the corresponding cell line**. Housekeeping GAPDH was used for intra sample normalization. Expression levels were normalized over the levels in K562S. Results are the average of three independent experiments ±SD. The statistical were calculated with two-tailed unpaired t-student’s t-test, and p-value of 0.05 was chosen as the limit of statistical significance.
